# Risk calculator of the clinical response to antihistamines in chronic urticaria: Development and internal validation

**DOI:** 10.1371/journal.pone.0295791

**Published:** 2024-02-23

**Authors:** Jorge Sánchez, Fabian Jaimes, Elizabeth García, Josefina Zakzuk, Ricardo Cardona, Margarita Velasquez

**Affiliations:** 1 Group of Clinical and Experimental Allergy, "IPS University" Clinic, University of Antioquia, Medellín, Colombia; 2 Internal Medicine Department, “San Vicente” Clinic, University of Antioquia, Medellín, Colombia; 3 Surgical Medical Unit-ENT (UNIMEQ-ORL), Bogotá, Colombia; 4 Health Economics Research Group, ALZAK Foundation, Immunological Research Institute, University of Cartagena, Cartagena, Colombia; 5 Dermatological Research Center, University of Antioquia, Medellín, Colombia; Universitas Indonesia Fakultas Kedokteran, INDONESIA

## Abstract

Early detection of CSU patients with low probability of a clinical response with antihistamines could undergo prompt initiation of therapeutic alternatives. The aim of the study was to develop and internally validate a model for predicting the clinical response to antihistamines in adult patients with chronic spontaneous urticaria (CSU), who consult allergology and dermatology care centers. A cohort of CSU patients, recruited from four participating centers, were followed up for 12 months. Fifteen candidate variables were selected to be included in the multivariate model and then internal validation was done with bootstrap analysis with 1000 simulations. The outcome variable, clinical response to antihistamines, was evaluated with the UAS (Urticaria Activity Score) scale for seven days: "No response to antihistamines" was defined as UAS7 ≥7 points after at least one month with a maximum dose of antihistamines, while "Response to antiH1" was defined as UAS7 ≤6 points for at least three months with the use of antiH1. A total of 790 patients were included. Among the different models analyzed, the model that included age, angioedema, anxiety/depression, time with the disease, NSAIDs (Non-steroidal anti-inflammatory drugs) intolerance, and UAS7 baseline was considered the one with the best performance (accuracy 0.675, HL 0.87, AUC 0.727). The internal validation analyses demonstrated good consistency of the model. In conclusion, this prediction model identifies the probability of response to antihistamines in patients with chronic spontaneous urticaria. The model could be useful for a personalized therapeutic approach according to individual patient risk.

## Introduction

Despite greater knowledge of chronic spontaneous urticaria (CSU) pathogenesis [1], the clinical evolution of this disease in each patient is currently uncertain. The first line of treatment in urticaria is the use of antihistamines (antiH1), but many patients (40%–60%), despite use for several months, do not achieve adequate clinical control with conventional or higher doses [2, 3]. In these cases, patients may benefit from the use of other therapies such as omalizumab or cyclosporine [4].

Multiple studies have attempted to use clinical and laboratory markers to predict the evolution of this disease in terms of its clinical response to treatment [5–18]; however, no single variable is sufficient to predict the clinical outcomes with antiH1 use. The development of a prediction model that includes a set of the most relevant variables may help to identify patients with a high probability of clinical response with antiH1 and patients with a low probability of response. Patients with a low probability of response could benefit from the early introduction of other therapies such as omalizumab or cyclosporine. We recently published a protocol [19] for the development and internal validation of a prediction model of the clinical response to antihistamines in patients with CSU. In this study, we present the results of this protocol, and discuss its clinical impact.

## Methods

### Study design and data source

We use a cohort design with 12 months of follow-up. The methodological details of the study were presented in an open access protocol [19]. Briefly, the main objective was the development and internal validation of a model [20, 21] and the reporting of its results according to the TRIPOD (Transparent Reporting of a Multivariable Prediction Model for Individual Prognosis or Diagnosis) statement [22–25] (S1 Table). Four centers located in two Colombian cities (Medellín and Bogotá) participated in the recruitment. The recruitment period was between 20-may-2020 to 26-Nov-2021, and the last patient follow-up until 04-Nov-2022. The predictive model was designed to be applied in patients with CSU who are going to receive continuous antiH1 treatment at conventional or high doses. Therefore, during the follow-up according to the medical indication, patients received antiH1 and then their clinical response was evaluated to determine the prognosis performance of the model three to four months later.

### Participants and eligibility criteria

Patients over 18 years with CSU were recruited based on international criteria [26]. Patients must have no contraindications to the use of antiH1 or comorbidities that could confound the diagnosis (e.g., hereditary angioedema, atopic dermatitis). As we describe in the protocol, at the time of entry into the study cohort, the patients must not be clinically controlled for their urticaria (UAS7 >7 points); This means that they had not yet received pharmacological treatment or that they had not received the maximum dose of antihistamines, since otherwise they would have to consider moving on to the next therapy (e.g., omalizumab). Once the patients were recruited, the information in the databases was deidentified for the authors and the work team to avoid possible biases.

### Primary outcome and predictors

The primary outcome was non-response with antiH1 at a doses four times higher than the conventional dose after at least one month of use. The clinical response was evaluated with the “Urticaria Activity Score” UAS7; “control” was defined as UAS7 less than or equal to 6 points (UAS7 ≤6). "No control" was defined as UAS7 greater than or equal to 7 points (UAS7 ≥7). Based on biological plausibility and previous comprehensive and systemic reviews [2, 27–31], we chose the following variables to be evaluated as part of the model [19]; sex, age, urticaria duration, atopy, angioedema, severity of symptoms, inducible urticaria, autoimmunity (define by an autoimmune disease), clinical history of intolerance to nonsteroidal anti-inflammatory drugs (NSAIDs), emotional disturbance (anxiety or depression), body mass index (BMI), C-reactive protein (CRP), anti-TPO IgG and anti-TPO IgE and, blood eosinophils counts. All candidate variables for the model were measured at baseline before starting antiH1 in conventional-dose or high-dose.

### Statistical analyses

Analyses were conducted using RStudio 4.2.1, JAMOVI 2.3.16, SPSS 26, and STATA 17. The sample size was calculated with 660 patients based on the criteria of events per variable [19, 32, 33] and an expected frequency of the outcome of 50%. The analysis, building, and development of the model was explained in detail in the protocol [19]. Briefly, the following steps were followed:

1) *Selection of candidate variables*: The selection of candidate variables for the model was made according to biological plausibility, an exhaustive review of the literature, and feasibility of its measurement in clinical practice [15, 28, 30, 31, 34–40]. Among the reasons for excluding some possible predictors was the difficulty of having these tests available during the first consultation (e.g., D dimer,), lack of consistency in previous studies regarding its association with the response to antihistamines (e.g., total IgE), and lack of availability in most clinical centers (e.g., Basophil Activation Test (BAT).
2) *Quality of the collected data and management of lost data*: In those variables with missing data of less than 10%, multiple imputation strategies were performed [19]. Variables that had a greater loss (>10%) were withdrawn.
3) *Data management*: Analysis of the collinearity assumption was conducted with a correlation matrix and by the variance inflation factor in continuous variables. For the categorical variables, a “chi-square test of independence” was used. The monotonic function relationship assumption was evaluated in the continuous variables graphically using the Lowess function; those variables that did not meet this assumption were transformed or dichotomized according to their smoothed function.
4) *Strategies to select the variables to include at the end of the model*: For each of the variables, the degree of association with the outcome variable was estimated both by the simple chi-square odds ratio (OR) and by a multiple logistic regression model. To select the independent variables associated with the outcome of interest, the Wald statistic was evaluated, considering *p* < 0.05 to include a variable as significant in the multivariate model. In addition, biological plausibility was considered as a criterion for variable inclusion.
5) *Evaluation of model performance with predictive accuracy and internal validation*: For selection of the best model, several models were built according to the inclusion and exclusion of variables. The discrimination and calibration of each model was evaluated to compare the predictive capacity of the models. Discrimination was evaluated graphically by the area under the curve of receiver operator characteristics (AUC-ROC) and using the c statistic, while calibration was evaluated with the Hosmer–Lemeshow hypothesis test. Other comparisons were carried out with the integrated discrimination index (IDI), the reclassification improvement index (NRI), the Akaike information criterion (AIC), and the Bayesian information criterion (BIC) [41, 42].

### Presentation format and clinical impact

The model is presented in an excel sheet for easy global access. The prediction model provides a risk estimate, where the lowest value presented as a percentage indicates a low risk, while the highest value indicates a high risk of "no response" with antiH1, even at four times the conventional dose. With the follow-up data after one year, we carried out an evaluation of the potential clinical impact of the model in the cohort of patients according to the decision-making that should be carried out based on the results of the final model.

### Ethical considerations

The research was approved by the Ethics committee of the University of Antioquia (CODE F-017-00) and has the institutional endorsement of each participating center. All patients signed the written informed consent before entering the study. Some patients participated in a previous study where information on some variables was collected, therefore they had signed two informed consents; one from the previous study and one for this study authorizing information from the previous study to be used in this research [19]. This project was funded by an inter-institutional public agreement (Hospital “Alma Mater de Antioquia”, University of Antioquia, Hospital “San Vicente Fundación”).

## Results

### Study population

**Fig 1:**
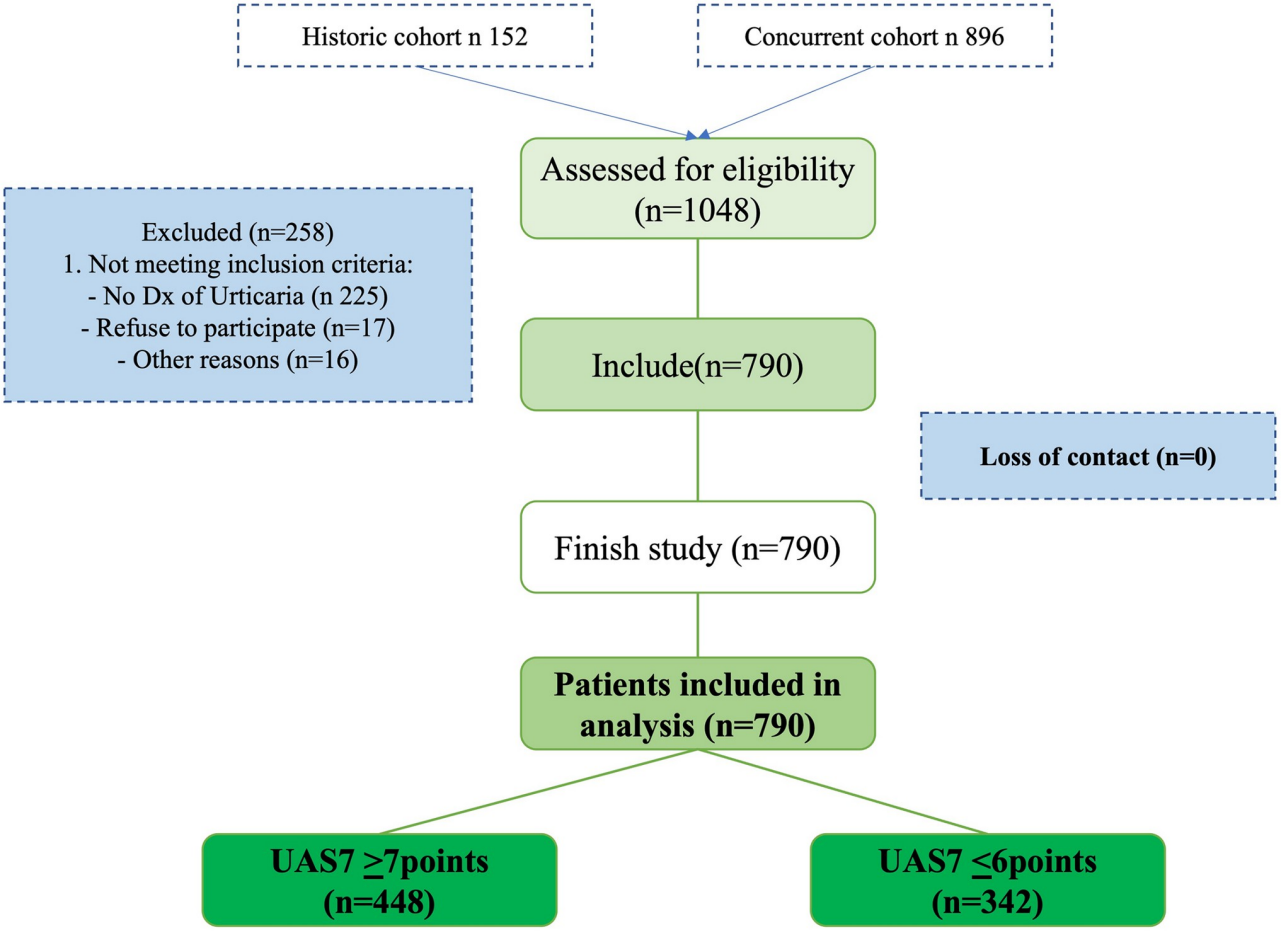
Flowchart of patient selection. Patients included and their relationship with the outcome. UAS7; Urticaria Activity Score for 7 days.

A total of 1048 patients were invited to participate (Fig 1), most of whom were from a concurrent cohort (*n* = 896) and some from a historic cohort (n 152). One hundred and six (11.8%) were excluded; they were initially referred to the study with the suspected diagnosis of CSU, but during recruitment this diagnosis was ruled out and other skin diagnosis were demonstrated. Of the historical cohort, data of the candidate variables were collected retrospectively, but patients still did not meet the time for the outcome, so they were followed up prospectively. The general characteristics of the population are presented in Table 1. Of the total number of patients included, 182 were from Bogotá and 608 from Medellín. We performed analyses comparing the predictor variables and the frequency of the outcome between the city of recruitment and the historical or concurrent data (S2 and S3 Tables). Among the centers in Bogotá, there was a lower score in the baseline UAS7 compared to the centers in Medellín (26 vs. 21), while those in Medellín had a higher body mass index (BMI) (21 vs. 25); however, these differences did not reach statistical significance or clinical minimum relevant difference from UAS7. We also did not observe differences in the frequency of the outcomes according to the different second-generation antiH1 used. There were no differences between the historical and concurrent data. All included patients at baseline (*n* = 790) completed the study and filled in the questionnaires, as well as supplied biological samples for the performance of paraclinical tests.

**Table 1:** General characteristics of the patients.

	Total patients n 790	UAS7 ≤6 n 342	UAS7 ≥7 n 448
**Patient characteristics**			
Female sex *	602 (76.2%)	255 (74.5%)	347 (77.4%)
Median age in years (SD range) *	30 years (SD 11.6 range 68)	32 (SD 13, range 68)	29 (SD 10, range 66)
*Age <49 years (%)*	735 (93%)	311 (90.9%)	424 (94.6%)
BMI *	25 (SD 3.2 range 27)	25 (SD 3.2 range 22.5)	25 (SD 3.3 range 27)
**Urticaria characteristics**			
CSU beginning (months)*	24 (SD 41.2 range 58.6)	21 (SD 50 range 58.6)	33 (SD 33 range 35.8)
CIU *	343 (43.4%)	91 (26.6%)	252 (56.2%)
Angioedema *	343 (43.4%)	91	252
UAS7 baseline *	26 (SD 7.8 range 35)	22 (SD 8 range 34)	26 (SD 7 range 35)
*UAS7 final*	10 (SD 10 range 42)	3 (SD 2 range 6)	18 (SD 8 range 35)
**Comorbidities**			
NSAIDs reaction*	102 (12.9%)	38 (11.1%)	64 (14.2%)
Anxiety / Depression *	252 (31.9%)	81 (23.6%)	171 (38.1%)
Autoimmune disease *	118 (14.9%)	53 (15.4%)	65 (14.5%)
**Paraclinical exams**			
Eosinophils *	124 (SD 133 range 1001)	125 (SD 141 range 1000)	124 (SD 126 range 1001)
Anti-TIPO IgG *	7.8 (SD 34.6 range 242)	7.5 (SD 33.9 range 211)	7.8 (SD 35.2 range 242)
C reactive protein *	0,3 (SD 1.3 range 14,2)	0.05 (SD 1.4 range 11.5)	0.05 (SD 1.25 range 14.2)
Atopy*	273 (34.6%)	117 (34.2%)	156 (34.8%)

Preselected variables for the prediction model are indicated with *. Median, range, and SD are presented because they do not have a normal distribution. UAS7 final; Represents UAS7 after using antihistamines at a conventional dose or four times the conventional dose. CIU: Chronic inducible urticaria.

### Characteristics of the predictor variables

The distribution of continuous variables was not normal (S1 Fig). It was not possible to measure anti-TPO IgE in all patients due to the COVID-19 pandemic (difficulties in obtaining reagents). Therefore, the percentage of missing data was 82.3%, and so it was not included in the prediction models. The other variables with missing data were anti-TPO IgG (*n* = 69, 8.7%), blood eosinophils (*n* = 72, 9.1%), and CRP (*n* = 76, 9.6%). For these three variables, the missing data were imputed.

There was no collinearity between the candidate variables for the model according to the different evaluation techniques used, and there was a monotonic relationship between the variables and the outcome (S2 Fig). Because age presented a turning point in the graph at 50 years, and this corresponds to the second epidemiological peak with the highest incidence of the disease, it was categorized into ≤49 and ≥50 years.

### Proposed models

The candidate variables were evaluated in relation to the outcome (Table 2). We developed four models (Table 3); the selection of variables for each model is described below:

**Model #1:** In the first model, we included all the proposed variables in their natural form as they were collected.
**Model #2:** We included all of variables in their natural form as they were collected, but with age transformed (categories ≤49 and ≥50 years).
**Model #3:** We chose the variables according to the pre-established criteria in the protocol for statistical significance.
**Model #4:** We chose variables based on statistical significance (variables in model #3) and biological plausibility: The female predominance suggests that there may be a different biological behavior by sex, and autoimmunity was included due to the probability of autoimmune diathesis, which would be in favor of greater chronicity and less of a response to pharmacological treatment with antihistamines.

**Table 2:** Statistical strategies to select variables.

Categorical variables	X^2^	Odds ratio
Sex	0.896 (p 0.344)	1.17 (0.843–1.63)
Chronic inducible urticaria	0.089 (p 0.785)	1.05 (0.773–1.42)
**Anxiety/Depression**	**18.7 (p <0.007)**	**1.99 (1.45–2.72)**
Autoimmunity	0.149 (p 0.6)	0.92 (0.62–1.37)
NSAIDs reactions	1.74 (p 0.1)	1.33 (0.86–2.06)
**Angioedema**	**69.4 (p <0.001)**	**3.55 (2.62–4.81)**
Atopy	0.032 (p 0.8)	1.03 (0.76–1.36)
**Age transformed**	**10 (p 0.001)**	**2.45 (1.99–3.01)**
**Continuous variables**	**p**	
Age	0.117	
**Urticaria duration**	**0.001**	
BMI	0.574	
**UAS7 baseline**	**<0.001**	
IgG anti-TPO	0.579	
Eosinophils	0.791	
PCR	0.576	

Statistical criteria depended on whether the variable was continuous (x^2^, Odds ratio) or categorical (Independent samples Mann-Whitney U test). NSAIDs: Non-steroidal anti-inflammatory drugs “Time”; Time from disease onset, “BMI”; the body mass index, “age transformed”; categories ≤49 and ≥50 years.

**Table 3:** Construction of different models.

Model #1: All variables	Model #2: All variables but age transformed	Model #3: statistically significance	Model #4: statistically significance plus biological plausibility
Age	Age (transformed)	Age (transformed)	Age (transformed)
Sex	Sex	//	Sex
Atopy	Atopy	//	//
Urticaria duration	Urticaria duration	Urticaria duration	Urticaria duration
Angioedema	Angioedema	Angioedema	Angioedema
CIU	CIU	//	//
Autoimmunity diseases	Autoimmunity diseases	//	Autoimmunity diseases
Anxiety and depression	Anxiety and depression	Anxiety and depression	Anxiety and depression
BMI	BMI	//	//
AINEs intolerance	AINEs intolerance	AINEs intolerance	AINEs intolerance
UAS7 at baseline	UAS7 at baseline	UAS7 at baseline	UAS7 at baseline
IgG anti-TPO	IgG anti-TPO	//	//
Blood eosinophils	Blood eosinophils	//	//
PCR	PCR	//	//

BMI; body mass index. CIU; inducible chronic urticaria. UAS7 Urticaria Activity Score. Time: Duration of the urticaria. CRP: C-reactive protein. // variable no included in the model.

### Selection of the best model

**Fig 2:**
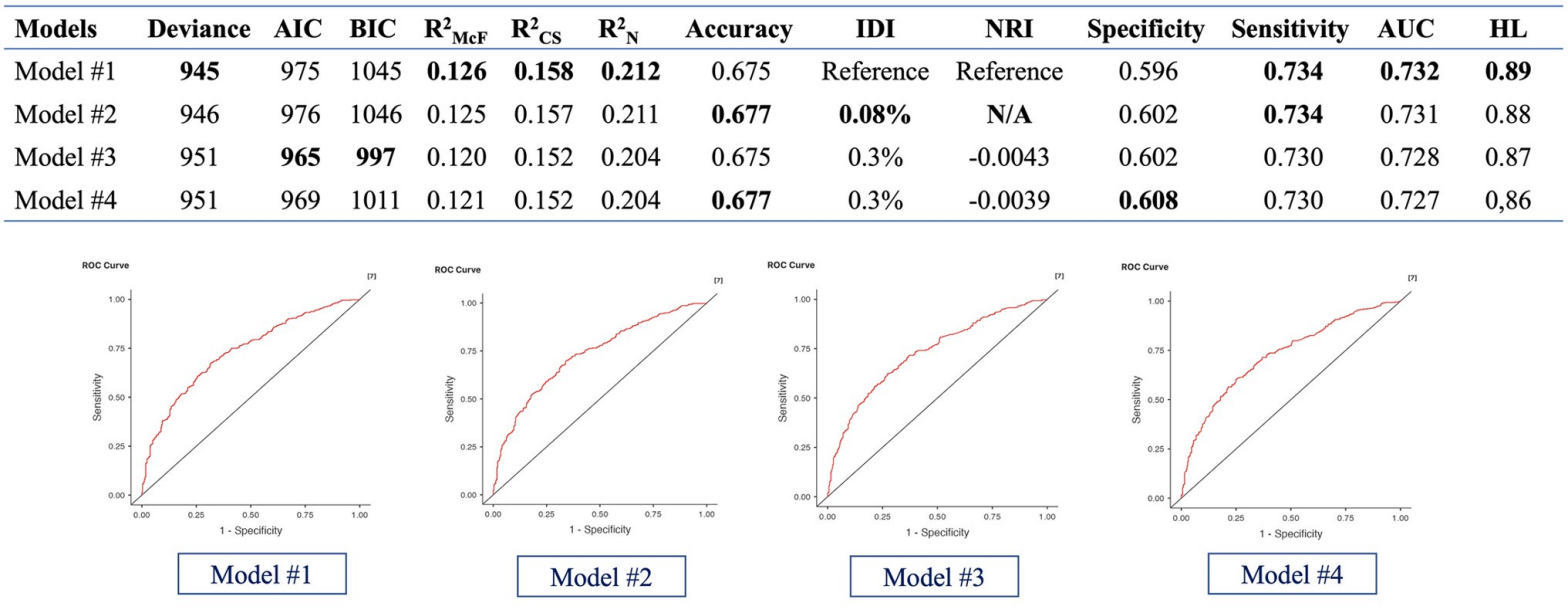
Comparison of the models. The models were compared according to different parameters. For the NRI, model 2 has a variance equal to model 1. AIC: Akaike Information Criterion. BIC: Bayesian Information Criterion. R^2^_McF_: R squared MacFadden. R^2^_CS_: R square Cox and Snell. R^2^_N_: R squared Negelkerke. IDI: Discrimination Index. NRI: Net Reclassification Improvement. N/A: Does not apply.

The performance of the four models is presented (Fig 2). All models had a calibration over 0.84 according to HL Hosmer-Lemeshow test, and a discrimination over 0.725 according to AUC. We used the IDI and the NRI index to evaluate if models 2, 3, and 4 had better performance than model 1, which included all the variables. Considering parsimony and easy applicability, we chose model 3 as the best model, since the four models had little difference in performance, but model 3 was the most parsimonious.

For the model, we applied the following mathematical model that expresses the probability of the event in question occurring as a function of the predictor variables: *P(Y = 1) = 1/1+exp(-B*_*0*_*-B*_*1*_*x*_*1*_*-B*_*1*_*x*_*2*_*-Bx*_*3*_*-B*_*4*_*x*_*4*_*-B*_*5*_*x*_*5*_*-B*_*6*_*x*_*6*_*)*

Correspondingly:

$$P\left(Y=1\right)=1/1+exp\left(-B_{intercept}-B_{Age}x_{1}-B_{Angiodema}x_{2}-B_{Anxiety\_Depressionn}x_{3}-B_{NSAIDs}x_{4}-B_{DurationUCE}x_{5}-B_{UAS7baselinel}x_{6}\right)$$

where B_0_ is the intercept and B_i_x_i_ is the coefficient of each variable, together with the result of that variable for the patient (x). Beta coefficients; Intercept -2.022, Age 0.116, angioedema 1, anxiety and depression 0.198, time 0.0001 (for each month), UAS7 baseline 0.062 (for each point).

In S4 Table, the final model chosen is presented in an Excel sheet. The presentation form provides a percentage estimate regarding the risk of not having a response with antihistamines even at doses four times the conventional one. For example, 0% represent a high probability of control and 100% represent a high probability of no clinical control with antiH1.

### Internal validation

As shown in Table 4, the selected final model after 1000 simulations with Bootstrap resampling did not show an important change in the standard error of the model variables, which suggests a good internal consistency of the model, and no additional adjustments were required.

**Table 4:** Internal validation.

	Development sample			Bootstrap	
	*B*	Standard Error	Sig.	Standard Error	Sig.
**Age trans**	*0*.*116*	0.328	0.722	0.361	0.775
**Time**	*0*.*001*	0.002	0.583	0,002	0.589
**Angioedema**	*1*.*193*	0.164	0.000	0.173	0.001
**Anxiety/Depression**	*0*.*624*	0.173	0.000	0.172	0.001
**AINEs**	*0*.*198*	0.237	0.404	0.235	0.397
**UAS7 baseline**	*0*.*062*	0.011	0.000	0.011	0.001
**Constant**	*-2*,*022*	0.391	0.000	0.440	0.001

Internal validation was performed using Bootstrap analysis with 1.000 simulations.

### Clinical implications and interpretation of the model

**Fig 3:**
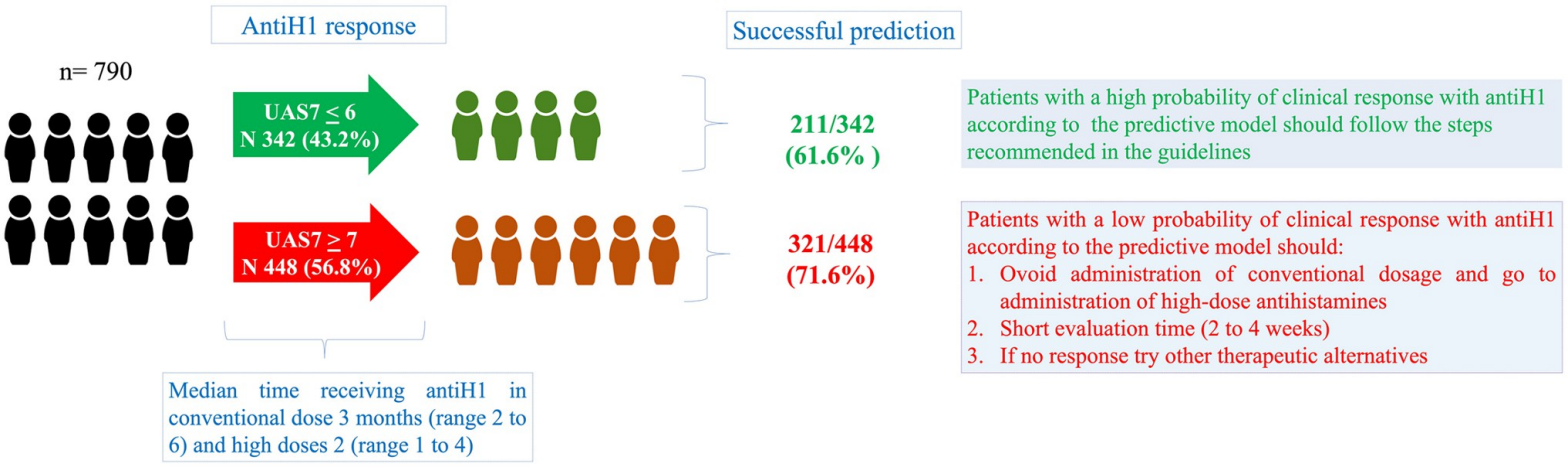
Clinical implications of the model. The predictive model would make it possible to identify patients with a higher risk of non-response to antihistamines and who would require prioritization in their management to reduce the time without clinical control.

The administration time of therapies (antihistamines, omalizumab, and cyclosporine) usually takes several months to evaluate the clinical response in patients (Fig 3). The model provides the probability of a patient having an unsatisfactory response to antihistamines from 0% (satisfactory response) to 100% (unsatisfactory response). the 50% point was useful for decision making with a good calibration: If the result of the model in a patient is <50%, we recommend following conventional management (conventional antiH1 dose and, if there is no response, an increase to a higher dose), and the control appointment can be after a few months. If the result is ≥50%, we suggest prioritizing patient care and avoiding a conventional antiH1 dose, instead administering the maximum dose of antihistamines immediately for one month. With these recommendations, it is possible to reduce the time of the therapeutic evaluation with antihistamines in patients with a low probability of a response and to ensure that they can access (if required) other therapies more quickly.

## Discussion

In CSU, although several variables (e.g., D-dimer, basophils, IgG anti-TPO) have been associated with the response to antiH1, none seem to have sufficient precision to predict the clinical response to this treatment [28, 31] and although several studies show a statistical association with outcomes of interest in urticaria, their diagnostic (sensitivity, specificity) or predictive (calibration, discrimination) performance has not been evaluated, which limits their clinical applicability. Some models have been developed to predict the response to omalizumab [43, 44], and some biomarkers have been proposed to predict the response to antH1 [16, 45]. However, to the best of our knowledge, this is the first risk calculator developed to predict the response to antiH1 in patients with CSU. Even in underdeveloped countries antiH1 are cheap and doing an empiric therapeutic trial to determine response is very simple and feasible. However, the time during the therapeutic trial can be from several weeks [46, 47] to months according to the availability of medical appointments and in an uncontrolled patient this has a high impact on their quality of life.

This research responds to a knowledge gap and a need in clinical practice; the guidelines support that antihistamines are useful in the management of urticaria [4] and evaluation of antiH1 response should be performed in eight weeks (four weeks for the conventional dose and four weeks for the higher dose) [4]. Nevertheless, 40%–60% of patients do not achieve adequate clinical control with this treatment [2, 48], and we observed that it took between 2 and 10 months to evaluate the therapeutic response (Fig 3). These results are like those observed in other countries, including the AWARE study that included cities from several Latin American and European countries [47, 48]. This generates an ethical conflict that is difficult to solve since the delay in medical care is because many medical centers always have full agenda, so control appointments are usually assigned after several months. Therefore, predicting the clinical response can help to promptly identify patients who could benefit from the use of alternative therapies, achieve early control of the disease, and avoid prolonged times when evaluating antiH1 therapeutic response. On the contrary, the model also makes it possible to identify patients with a high probability of a good response to antihistamines, which corresponds to patients who will not require other alternatives therapies. In these patients, the assignment of an appointment can be more flexible, which allows reducing the burden of consultations in terms of both the health system and patients’ time. In order to make this prediction quickly and allow the most efficient management for the patient, it is necessary to include in the prediction model variables that are easily available from the first consultation, so laboratory variables (e.g., D- dimer, eosinophils, PCR, basophils, etc.) could be less useful as they are not always available from the first consultation. However, these variables should be considered in other prediction models where the outcome does not require immediate decision-making, such as the duration of urticaria or for define its endotype [13, 15].

The prediction model helps to identify those patients who are candidates for omalizumab and/or cyclosporine, which constitute the next management step when there is no satisfactory clinical response to antihistamines. Omalizumab is a treatment that has been shown to be effective in the management of urticaria [2, 43, 49], with 40%–60% of patients who do not respond to antihistamines showing a satisfactory response (UAS7 < 6) to omalizumab and an additional 20%–30% achieving complete control (UAS7 = 0). The effectiveness of cyclosporine appears to be similar, although its safety profile is lower [2, 50, 51]. However, these therapies are expensive, especially omalizumab, so the model provides an additional clinical tool to justify the individualized use of these therapies in patients who require it.

The development and validation of the model has several strengths; the sample size was higher than expected and there were no lost cases. The number of events was close to what was predicted in the design, and the consistency in the internal validation was adequate. Only one variable had to be removed from the model, and data losses were few. The final model is made up of six simple variables (age, duration, angioedema, anxiety/depression, NSAID intolerance, and UAS7) that, in previous studies, have been associated with severity, duration, and lack of response to drug treatments [16, 28, 52]. The variable that had the greatest impact in the model was the activity of the disease evaluated in the UAS7. To use this variable in the patient’s first medical visit, we asked about the number of hives and pruritus in the last week previous of the visit (see S4 Table). In this way, we can avoid having to make follow-up appointments or calls that could hinder the applicability of the predictive model. The variables of the model are easy to collect, affording the model accessibility for practically any patient or level of care. However, patients in this study were cared for by urticaria specialists, so it is required in the future to evaluate its transportability for use by primary care physicians.

Another strength of the study is that it was multicenter, including health centers in different countries. We did not consider carrying out multilevel analysis, since previous studies carried out in these populations did not indicate that it was necessary [9, 27]. Most of the recruited patients were in the city of Medellín, but when comparing the patients between the two participating cities, we did not observe important differences between the characteristics of the populations, the frequency of the variables, or the outcomes. There are multiple second-generation antihistamines and several meta-analyses have been performed, including studies that have compared their efficacy [2, 39]. In our study, patients received loratadine, cetirizine, levocetirizine, desloratadine, fexofenadine, and bilastine. In some patients, the antiH1 used at the conventional dose was different from that used at a high dose. The type of antihistamine used did not affect the performance of the predictive model, which allows the predictive model to be used regardless of the type of antihistamine that the patient is going to use.

A weakness of the model is its moderate performance. For prediction models, the parameters of greatest interest are different from those frequently used to evaluate a diagnostic test [53]. The model that we chose had satisfactory calibration (HL: 0.87), discrimination (AUC: 0.728), but accuracy was moderate (0.675). In a practical way, the model could be interpreted as correctly identifying 73% of patients who will not have clinical control with antihistamines and 60% of patients who will respond to treatment. This generates some problems at the time of decision making, since the usefulness of the model may or may not be adequate depending on the physician or health system perspective. From the point of view of the physician, the model is useful, since a high number of patients who will require new therapies are identified early; on the other hand, from the perspective of the health payer, the model would imply a 25% increase in patients who would erroneously receive high-cost therapies. Nevertheless, with some recommendations, we consider that the model can be useful in decision making, avoiding additional costs for the health system: According to the prediction model, patients with a low probability of clinical response should receive a high dose of antihistamines, omitting the conventional dose and prioritized care to promptly assess the response to the antihistamine. On the contrary, patients with a high probability of response could be given a conventional dose, and the follow-up period could be less urgent. To evaluate the additional expense that could be generated using the predictive model with these recommendations for the health system, we carried out a sensitivity analysis and the additional expense in the worst scenario would be only 3%. Therefore, we consider that the model is useful for the patient, the physician and with the recommendations that we did before does not generate a significant additional expense for health systems.

Another potential weakness of the model from the clinical point of view is that we use disease activity as the outcome evaluation scale. This scale does not consider the patient’s perception of control as the “urticaria control test” does [4]; however, both scales present a moderate correlation: In our population, it was 0.748, so in general, adequate control on one scale reflects good control over the other. Another point that could limit the performance of the model is that we include patients with a mean disease of 24 months; this model could be most useful in patients who recently start with urticaria; however, according to previous studies such as AWARE [46, 47], this is the time a patient takes to attend a specialized urticaria centers even in Europe, and unfortunately in more than 60% of cases it does not arrive with adequate management. Therefore, our predictive model is useful among specialists, but in future, it is necessary to carry out an external validation that allows to evaluate the reproducibility of the model, its performance in other populations, and its transportability to primary care physicians.

In conclusion, this model can be useful for the application of precision medicine in clinical practice to identify patients who are less likely to achieve control with antihistamines and prioritize their care as the start of the maximum dose of antihistamine.

## Supporting information

S1 Table Reporting of results according to the TRIPOD statement. (PDF)

S2 Table Variables according to the city of recruitment. (DOCX)

S3 Table Variables according to the type of recruitment. (DOCX)

S4 Table Final model. (XLSX)

S1 Fig Distribution of variables. The distribution of continuous variables was evaluated using the Shapiro Wilks test (A); with a *p* ≤0.05 we reject the null hypothesis that the distribution is normal. We also presented graphically the distribution of continuous variables (B).
(TIF)

S2 Fig Collinearity and monotone relation. We evaluated collinearity using the variance inflation factor (VIF) (A) and a correlation matrix (B). None of the variables presented a clear monotonic relationship (C).
(TIF)

S1 Data (XLSX)

S1 Graphical abstract (TIF)
