# Supplementary material for: Risk calculator of the clinical response to antihistamines in chronic urticaria: Development and internal validation
Source: PLoS One. 2024 Feb 23;19(2):e0295791. doi: 10.1371/journal.pone.0295791 (PMC10889609; doi:10.1371/journal.pone.0295791)
Supplement: S2 Table — (DOCX) [file pone.0295791.s002.docx]

**S2 Table. Variables according to the city of recruitment.**

|  | **Total patients (n 790)** | **Medellin (n 608)** | **Bogotá (n 182)** |
| --- | --- | --- | --- |
| **Patient characteristics** |  |  |  |
| Female sex * | 602 (76.2%) | 471 (77.5%) | 131 (71.6%) |
| Age (median) * | 30 years (SD 11.6 range 68) | 30 (SD 11,3, range 68) | 31 (SD 12.6, range 65.4) |
| BMI * | 25 (SD 3.2 range 27) | 25 (SD 3,11, range 22.5) | 21 (SD 8.4, range 34) |
| **Clinical features** |  |  |  |
| CSU beginning (months)* | 24 (SD 41.2, range 58.6) | 24 (SD 42 range 58.6) | 25 (SD 38.5, range 29.8) |
| CIU * | 343 (43.4%) | 187 (30.8%) | 60 (32.8%) |
| Angioedema * | 343 (43.4%) | 260 (42.8%) | 83 (45.4%) |
| UAS7 baseline * | 26 (SD 7.8, range 35) | 26 (SD 7.5, range 35) | 21 (SD 8.4, range 34) |
| UAS7 p4 | 10 (SD 10, range 42) | 12 (SD 9.9, range 42) | 6 (10.3, range 42) |
| **Comorbidities** |  |  |  |
| NSAIDs reaction* | 102 (12.9%) | 75 (12.3%) | 27 (14.8%) |
| Anxiety / Depression * | 252 (31.9%) | 190 (31.3%) | 62 (33.9%) |
| Autoimmune disease * | 118 (14.9%) | 97 (16%) | 21 (11.5%) |
| **Paraclinical test** |  |  |  |
| Eosinophils * | 124 (SD 133, range 1001) | 126 (SD 132, range 900) | 123 (SD 155, range 1001) |
| Anti-TIPO IgG * | 7.8 (SD 34.6, range 242) | 8 (SD 36, range 242) | 6 (SD 31.3, range 242) |
| C reactive protein * | 0,05 (SD 1.3, range 14,2) | 0.05 (SD 1.2, range 14.2) | 0.04 (SD 1.5, range 11.6) |
| Atopy* | 273 (34.6%) | 213 (35%) | 60 (32.8%) |

**S2 Table**. reselected variables for the prediction model are indicated with *. Median, range, and SD are presented because they do not have a normal distribution. UAS7 final; Represents UAS7 after using antihistamines at a conventional dose or four times the conventional dose. CIU: Chronic inducible urticaria.
