# Supplementary material for: Risk calculator of the clinical response to antihistamines in chronic urticaria: Development and internal validation
Source: PLoS One. 2024 Feb 23;19(2):e0295791. doi: 10.1371/journal.pone.0295791 (PMC10889609; doi:10.1371/journal.pone.0295791)
Supplement: S3 Table — (DOCX) [file pone.0295791.s003.docx]

**S3 Table. Variables according to the type of recruitment.**

|  | **Total patients (n 790)** | **historic (n 152)** | **Concurrent (n 638)** |
| --- | --- | --- | --- |
| **Patient characteristics** |  |  |  |
| Female sex * | 602 (76.2%) | 118 (77.6%) | 484 (75.8%) |
| Age (median) * | 30 years (SD 11.6, range 68) | 30 years (SD 12.2, range 69) | 30 years (SD 11.4, range 68) |
| Age <49 years | 735 (93%) | 137 (90.1%) | 598 (93.7%) |
| BMI * | 25 (SD 3.2, range 27) | 24 (SD 3, range 27) | 25 (SD 3.8, range 27 |
| **Urticaria characteristics** |  |  |  |
| CSU beginning (months) | 24 (SD 41.2, range 58.6) | 20 (SD 41.2, range 55.6) | 22 (SD 39.2, range 59.0) |
| CIU * | 343 (43.4%) | 67 (44%) | 276 (43.2%) |
| Angioedema * | 343 (43.4%) | 65 (42.7%) | 278 (43.5%) |
| UAS7 baseline * | 26 (SD 7.8, range 35) | 25 (SD 9.8, range 35) | 2 7 (SD 10.8, range 38) |
| UAS7 p4 | 10 (SD 10, range 42) | 10 (SD 10, range 42) | 10 (SD 10, range 42) |
| **Comorbidities** |  |  |  |
| NSAIDs reaction* | 102 (12.9%) | 20 (13.1%) | 82 (12.8%) |
| Anxiety / Depression * | 252 (31.9%) | 50 (32.8%) | 202 (31.6%) |
| Autoimmune disease * | 118 (14.9%) | 25 (16.4%) | 93 (14.5%) |
| **Paraclinical tests** |  |  |  |
| Eosinophils * | 124 (SD 133, range 1001) | 12(SD 130, range 981) | 124 (SD 140, range 1111) |
| Anti-TIPO IgG * | 7.8 (SD 34.6, range 242) | 8.7 (SD 33.3, range 242) | 7.2 (SD 36.4, range 232) |
| C reactive protein * | 0.05 (SD 1.3, range 14,2) | 0.07 (SD 0.9, range 14,8) | 0.05 (SD 1.4 range 13,8) |
| Atopy* | 273 (34.6%) | 54 (35.5%) | 219 (34.3%) |

**S3 Table**. reselected variables for the prediction model are indicated with *. Median, range, and SD are presented because they do not have a normal distribution. UAS7 final; Represents UAS7 after using antihistamines at a conventional dose or four times the conventional dose. CIU: Chronic inducible urticaria.
