# Supplementary figures and images for: Risk calculator of the clinical response to antihistamines in chronic urticaria: Development and internal validation
Source: PLoS One. 2024 Feb 23;19(2):e0295791. doi: 10.1371/journal.pone.0295791 (PMC10889609; doi:10.1371/journal.pone.0295791)

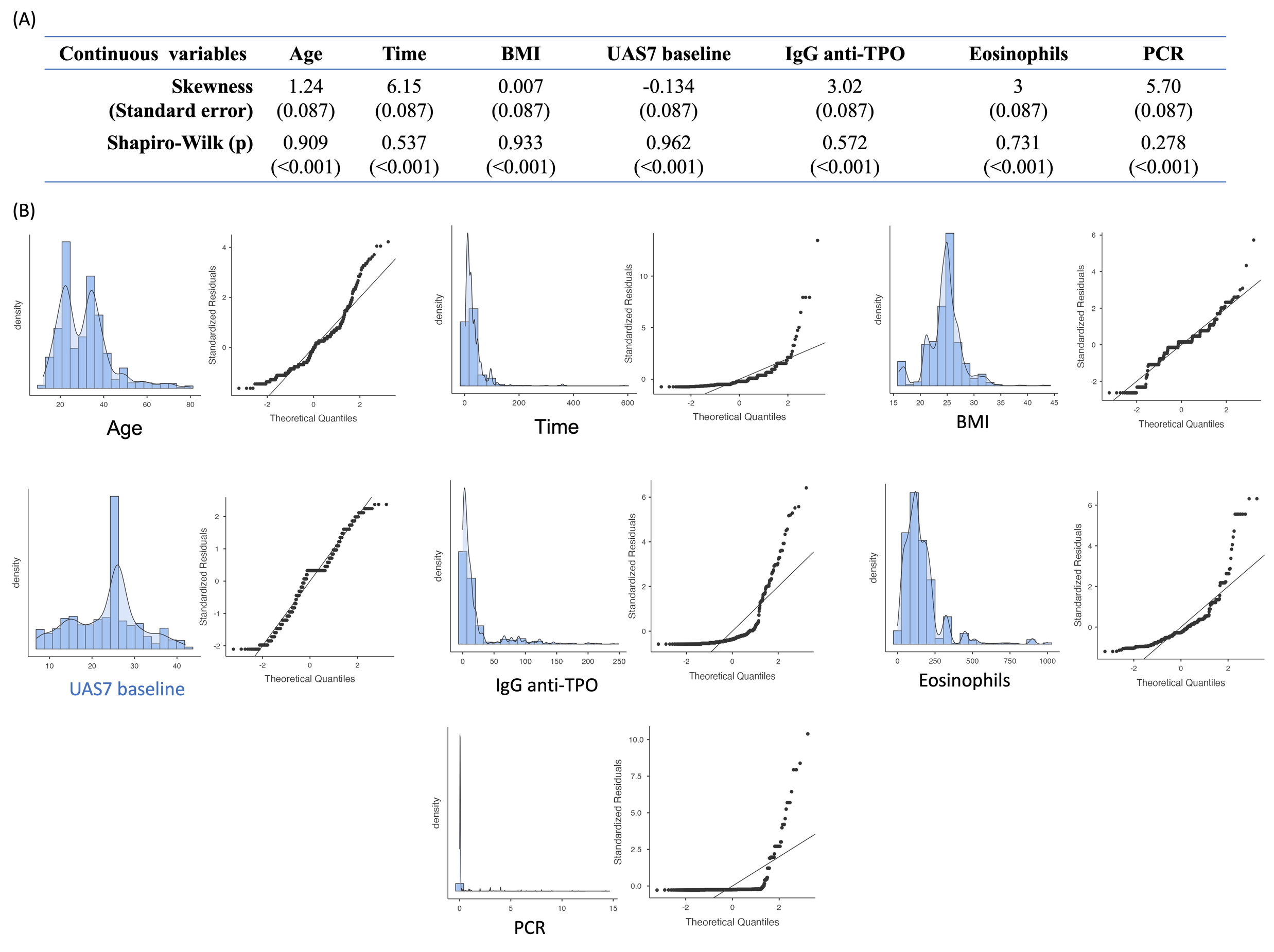

Supplement: S1 Fig — The distribution of continuous variables was evaluated using the Shapiro Wilks test (A); with a p ≤0.05 we reject the null hypothesis that the distribution is normal. We also presented graphically the distribution of continuous variables (B). (TIF) [file pone.0295791.s005.tif]

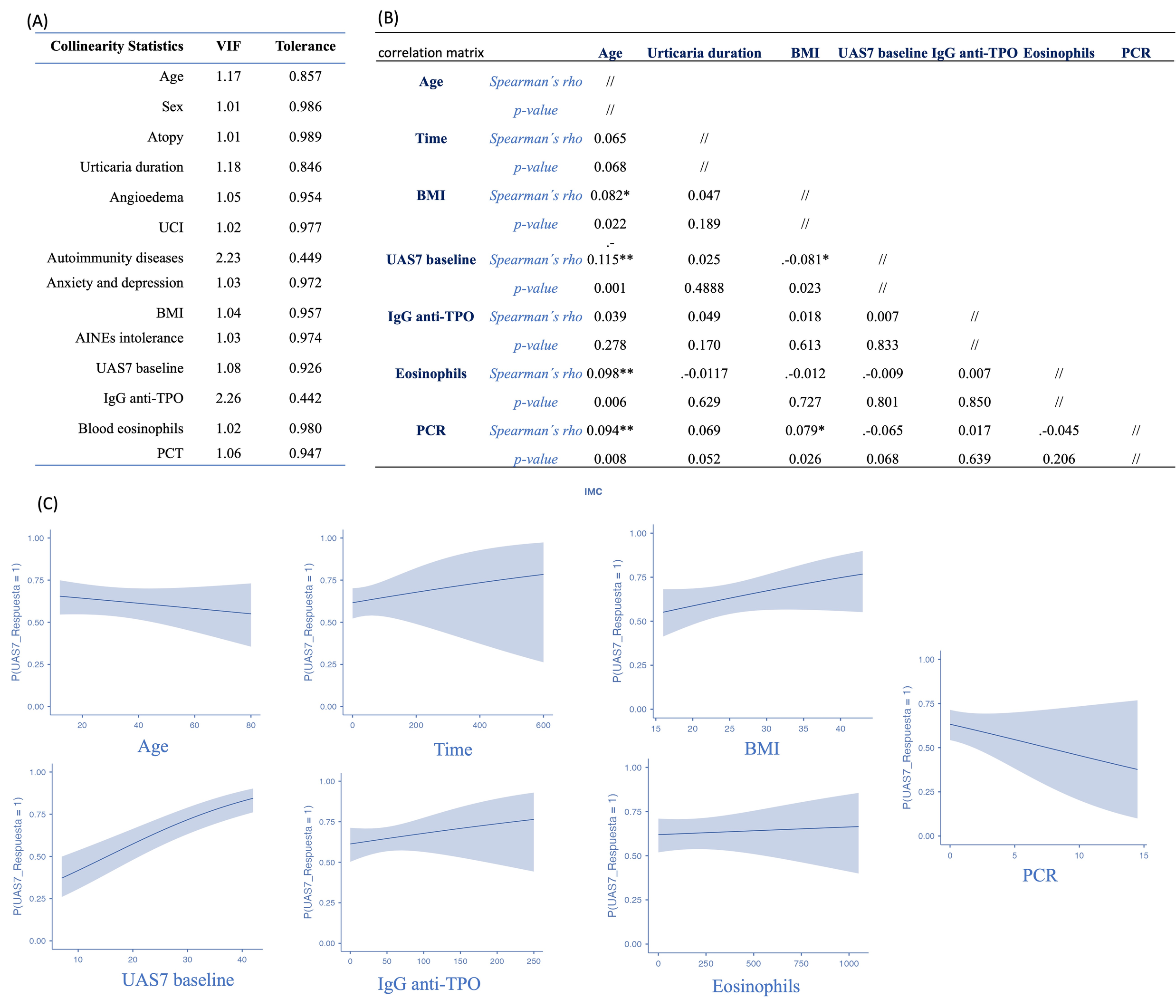

Supplement: S2 Fig — We evaluated collinearity using the variance inflation factor (VIF) (A) and a correlation matrix (B). None of the variables presented a clear monotonic relationship (C). (TIF) [file pone.0295791.s006.tif]

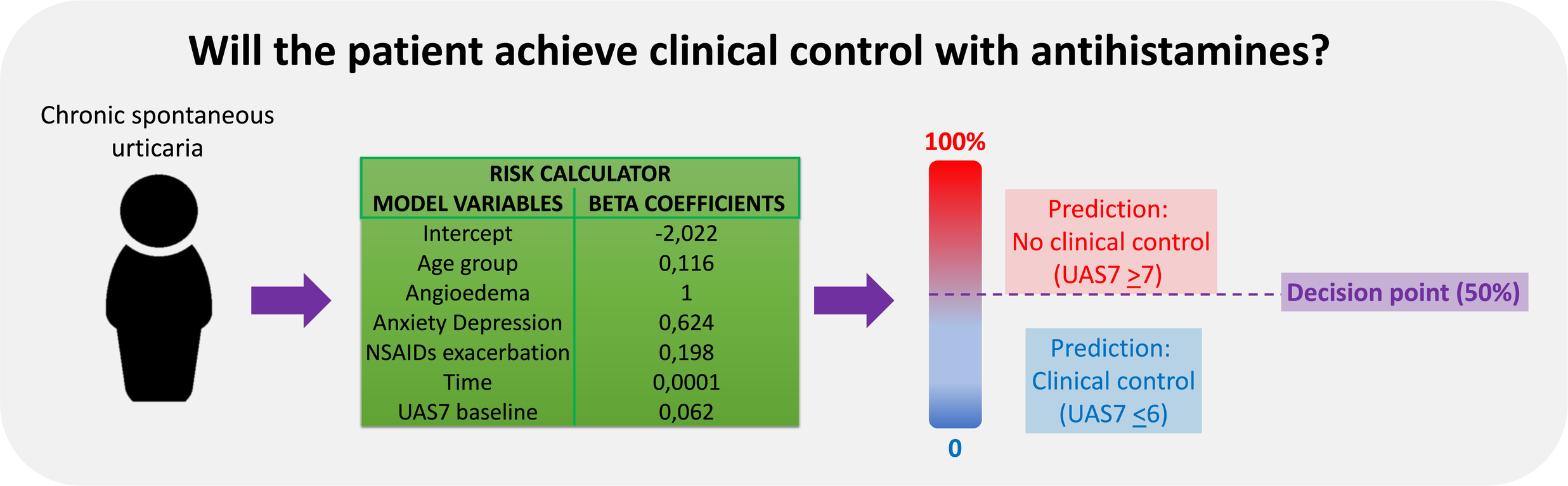

Supplement: S1 Graphical abstract — (TIF) [file pone.0295791.s008.tif]
